# Supplementary figures and images for: Nutritional status and its associated factors among commercial female sex workers in Hawassa city, south Ethiopia
Source: PeerJ. 2023 Apr 28;11:e15237. doi: 10.7717/peerj.15237 (PMC10150714; doi:10.7717/peerj.15237)

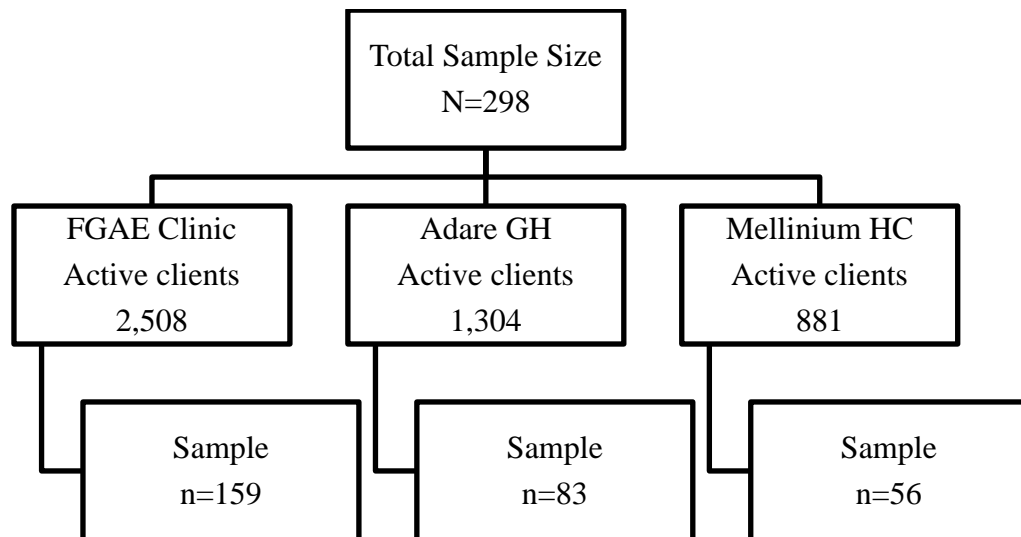

Supplement: Supplemental Information 1 [file peerj-11-15237-s001.pdf]
