# Supplementary material for: Nutritional status and its associated factors among commercial female sex workers in Hawassa city, south Ethiopia
Source: PeerJ. 2023 Apr 28;11:e15237. doi: 10.7717/peerj.15237 (PMC10150714; doi:10.7717/peerj.15237)
Supplement: Supplemental Information 2 [file peerj-11-15237-s002.docx]

| Name of the facility | Year service started | Clients ever enrolled | Clients served in 2021 | Weekly client visits (a) | Estimated client for 8 weeks (b=8*a) | Sampling fraction (n) | Interval  K = b/n |
| --- | --- | --- | --- | --- | --- | --- | --- |
| FGAE Confidential Clinic | 2012 | 12,360 | 2,508 | 58 | 464 | 159 | 2.9th |
| Adare KP Clinic | 2019 | 1,980 | 1,304 | 31 | 248 | 83 | 3.0th |
| Millennium KP Clinic | 2020 | 1,555 | 881 | 16 | 128 | 56 | 2.3th |
| Total |  | 15,895 | 4,693 |  |  |  |  |
