# Supplementary material for: Nutritional status and its associated factors among commercial female sex workers in Hawassa city, south Ethiopia
Source: PeerJ. 2023 Apr 28;11:e15237. doi: 10.7717/peerj.15237 (PMC10150714; doi:10.7717/peerj.15237)
Supplement: Supplemental Information 3 [file peerj-11-15237-s003.docx]

| Factors | | BMI (kg/m^2^) | | | Chi-square  *X*^2^(df, N=297)  *P* value |
| --- | --- | --- | --- | --- | --- |
|  |  | Underweight  (n=42) | Normal  (n= 205) | Overweight/ Obese (n=50) |  |
| Age (years) | |  |  |  | *X*^2^(4, 297) = 41.59  P < 0.001 |
|  | < 25 | 25 (18.0) | 109 (78.4) | 5 (3.6) |  |
|  | 25-29 | 7 (6.0) | 76 (65.5) | 33 (28.4) |  |
|  | >= 30 | 10 (23.8) | 20 (47.6) | 12 (28.6) |  |
| Having other work | |  |  |  |  |
|  | Yes | 25 (23.1) | 78 (72.2) | 5 (4.6) | *X*^2^(2, 297) = 25.01 |
|  | No | 17 (9.0) | 127 (67.2) | 45 (23.8) | P < 0.001 |
| Average daily income | |  |  |  |  |
|  | < 500 Birr | 24 (19.0) | 77 (61.1) | 25 (19.8) | *X*^2^(2, 297) = 6.89 |
|  | >= 500 Birr | 18 (10.5) | 128 (74.9) | 25 (14.6) | P = 0.032  *X*^2^(2, 297) = 17.75 |
| Years of experience | |  |  |  |  |
|  | < 4 years | 33 (17.2) | 139 (72.4) | 20 (10.4) |  |
|  | >= 4 years | 9 (8.6) | 66 (62.9) | 30 (28.6) | P < 0.001 |
| With whom living with | |  |  |  |  |
|  | Alone | 5 (4.7) | 80 (75.5) | 21 (19.8) | *X*^2^(4, 297) = 16.68 |
|  | Children/husband | 10 (20.4) | 27 (55.1) | 12 (24.5) | P = 0.002 |
|  | Other CSWs | 27 (19.0) | 98 (69.0) | 17 (12.0) |  |
| Drink alcohol regularly | |  |  |  |  |
|  | Yes | 29 (12.3) | 171 (72.5) | 36 (15.3) | *X*^2^(2, 297) = 6.46 |
|  | No | 13 (21.3) | 34 (55.7) | 14 (23.0) | P = 0.04 |
| Chew Khat regularly | |  |  |  |  |
|  | Yes | 23 (10.6) | 158 (72.5) | 37 (17.0) | *X*^2^(2, 297) = 8.90 |
|  | No | 19 (24.1) | 47 (59.5) | 13 (16.5) | P = 0.012 |
| Use substances/drugs regularly | |  |  |  |  |
|  | Yes | 27 (30.3) | 56 (62.9) | 6 (6.7) | *X*^2^(2, 297) = 31.95 |
|  | No | 15 (7.2) | 149 (71.6) | 44 (21.2) | P < 0.001  *X*^2^(2, 297) = 30.53 |
| Use drugs in exchange of sex | |  |  |  |  |
|  | Yes | 15 (45.5) | 13 (39.4) | 5 (15.2) |  |
|  | No | 27 (10.2) | 192 (72.7) | 45 (17.0) | P < 0.001  *X*^2^(2, 297) = 8.28 |
| Use mobile for catching clients | |  |  |  |  |
|  | Yes | 21 (10.2) | 148 (72.2) | 36 (17.6) |  |
|  | No | 21 (22.8) | 57 (62.0) | 14 (15.2) | P = 0.016 |
| Usual place of sex | |  |  |  |  |
|  | Hotel/home based | 9 (5.4) | 113 (67.7) | 45 (26.9) | *X*^2^(2, 297) = 43.94 |
|  | Street based | 33 (25.4) | 92 (70.8) | 5 (3.8) | P < 0.001  *X*^2^(2, 297) = 10.18  P = 0.006 |
| Use any modern contraceptives | |  |  |  |  |
|  | Yes | 27 (11.1) | 173 (71.2) | 43 (17.7) |  |
|  | No | 15 (27.8) | 32 (59.3) | 7 (13.0) |  |
| HIV status | |  |  |  |  |
|  | Positive | 24 (48.0) | 18 (36.0) | 8 (16.0) | *X*^2^(4, 297) = 58.26 |
|  | Negative | 18 (7.3) | 187 (75.7) | 42 (17.0) | P < 0.001 |
| Presence of any chronic illness | |  |  |  |  |
|  | Yes | 12 (23.1) | 18 (34.6) | 22 (42.3) | *X*^2^(4, 297) = 38.67 |
|  | No | 30 (12.2) | 187 (76.3) | 28 (11.4) | P < 0.001 |
