# Supplementary material for: Nutritional status and its associated factors among commercial female sex workers in Hawassa city, south Ethiopia
Source: PeerJ. 2023 Apr 28;11:e15237. doi: 10.7717/peerj.15237 (PMC10150714; doi:10.7717/peerj.15237)
Supplement: Supplemental Information 6 [file peerj-11-15237-s006.docx]

# **QUESTIONNAIRE (English version)**

1. **Quantitative Survey**   **Code: _________**

**Section I: Socio-demographic and economic characteristics**

| **ID** | **Questions** | **Possible Answers** | **Skip** |
| --- | --- | --- | --- |
| 101 | Present Age | __________ years |  |
| 102 | Lived in the past | 1. Rural 2. Urban |  |
| 103 | Educational status of respondent | 1. No education 2. Primary education 3. Secondary education 4. College and above |  |
| 104 | What is/was your religion? | 1. Orthodox 2. Muslim 3. Protestant 4. If any other, specify ______ |  |
| 105 | Do you have any other work than sex work? | 1. Yes 2. No …………………………> | Q107 |
| 106 | If yes to Q105, what do you work? | 1. Waitress 2. Broker agent 3. Small business 4. If any other, specify _______ |  |
| 107 | What is your marital status now? | 1. Single 2. Married 3. Widowed 4. Divorced/separated/abandoned |  |
| 108 | Do you have a child or children? | 1. Yes 2. No …………………………> | Q110 |
| 109 | Do you have under 5 years of age child/ren? | 1. Yes 2. No |  |
| 110 | Who takes care of young children when you left home? | 1. No one 2. My family 3. Older children 4. Any other, specify |  |
| 111 | If yes to Q109, how many children do you have? | ___________ |  |
| 112 | How much is your daily income? | ____________ Birr |  |
| 113 | How much is your daily expenditure? | _____________ Birr |  |

**Section II: Behavioral/lifestyle factors including risky sexual behaviors**

| **ID** | **Questions** | **Possible Answers** | **Skip** |
| --- | --- | --- | --- |
| 201 | What is the cause of commercial sex work? | 1. Lower economy 2. Sexual harassment 3. Exploitation 4. Peer pressure 5. If any, specify ______ |  |
| 202 | How many days did you work for sex work last week? | ________ days |  |
| 203 | Age entering into commercial sex work/first sexual debut? | ________ years |  |
| 204 | How many years of experience did you have in sex business) | ________ years |  |
| 205 | Whom are you living with? | 1. Own 2. Husband 3. Children 4. Other CSWs 5. If any other, specify _______ |  |
| 206 | Do you currently smoke? | 1. Yes 2. No 3. I used to smoke |  |
| 207 | Do you drink alcohol? | 1. Yes 2. No |  |
| 208 | Do you chew Khat? | 1. Yes 2. No |  |
| 209 | Do you use substance/drug? | 1. Yes 2. No ……………………….> | Q212 |
| 210 | If yes to Q209, what type of substance/drug do you use? | 1. Cannabis/powder 2. Injection 3. ‘Shisha’ 4. Ganja 5. If any other, specify _______ |  |
| 211 | Did you take drug in exchange of sex work from clients? | 1. Yes 2. No |  |
| 212 | Did you take ‘drug’/alcohol before sex work? | 1. Yes 2. No |  |
| 213 | What is your usual mass media exposure? | 1. Nothing 2. TV/Radio/FM 3. Social media (Facebook, telegram etc) 4. If any other, specify ________ |  |
| 214 | Do you use mobile for catching clients? | 1. Yes 2. No |  |
| 215 | Are you under ‘pimp’ via women get client through? | 1. Yes 2. No |  |
| 216 | Where is your usual place of sex business? | 1. Hotel based 2. Home based 3. Street based 4. If any other, specify _______ |  |
| 217 | Who are your usual clients? | 1. Businessman 2. Drivers (truck, bus, bajaj etc) 3. Gov’t worker 4. Students 5. I don’t know 6. If any other, specify _______ |  |
| 218 | How many clients per day do you have on average? | ____________ |  |
| 219 | How many clients’ turnover do you have last week? | ____________ |  |
| 220 | Do you take adequate time interval between two coitus? | 1. Yes 2. No |  |
| 221 | How many coital frequencies per client on average? | ___________ |  |
| 222 | Do you have non-paying sexual partner/lover/boy-friend? | 1. Yes 2. No |  |
| 223 | Do you have ever experienced in group sex? | 1. Yes 2. No |  |
| 224 | Do you have ever experienced anal sex? | 1. Yes 2. No |  |
| 225 | Do you have ever experienced sex with same sex? | 1. Yes 2. No |  |
| 226 | Do you have ever experienced masturbation? | 1. Yes 2. No |  |
| 227 | Do you watch pornography regularly? | 1. Yes 2. No |  |

**Section III: Health-hygiene Practices**

| **ID** | **Questions** | **Possible Answers** | **Skip** |
| --- | --- | --- | --- |
| 301 | Do you use condom during sex? | 1. Yes, consistently 2. Yes, but use irregularly 3. I don’t use with non-paying, permanent clients only 4. Not use |  |
| 302 | What is the source of condom? | 1. Hotel 2. Government facilities 3. NGO clinics 4. Self-buying 5. If any, specify __________ |  |
| 303 | Currently, do use any modern contraceptive methods? | 1. Yes 2. No ……………………….> | Q.306 |
| 304 | If yes to Q303, what type of modern contraceptive methods do you use? | 1. Pills 2. Injectable 3. Implants 4. IUD 5. If any other, specify _______ |  |
| 305 | Did you have any history of abortion in the past? | 1. Yes 2. No ……………………….> | Q.309 |
| 306 | If yes to Q306, how many incidences of abortion do you get? | ___________ |  |
| 307 | If yes to Q306, did you get any medical treatment for the abortion? | 1. Yes 2. No, manage myself ……..> | Q.309 |
| 308 | If yes to Q308, from where did you get medical treatment or procedure for the abortion? | 1. Gov’t health facility 2. Private/NGO health facility 3. If any other, specify _______ |  |
| 309 | Do you have access soap and water after sex at workplaces? | 1. Yes 2. No |  |
| 310 | Do you douche with soap and water after-coitus? | 1. Yes 2. No |  |
| 311 | Did you have any history of STIs diagnosis? | 1. Yes 2. No ………………………> 3. Don’t know ……………..> | Q.314  Q.314 |
| 312 | If yes to Q312, have you got treated for STIs? | 1. Yes 2. No |  |
| 313 | Currently, do you have any sign or symptom of smelly genital discharge/ulcer, lower abdomen pain without menstruation? | 1. Yes 2. No |  |
| 314 | What is your HIV status? | 1. Positive 2. Negative …………………> 3. Don’t know ……………...> | Q.318  Q.318 |
| 315 | If HIV status is positive, have you ever started ART? | 1. Yes 2. No ……………………….> | Q.318 |
| 316 | If ever started ART, are you currently on ART? | 1. Yes 2. No |  |
| 317 | When did you tested for HIV the last time? | 1. In last three months 2. In last one year 3. More than one year |  |
| 318 | Have you ever diagnosed for any other medical problems? | 1. Yes 2. No ………………………> | End P.III |
| 319 | If yes to Q318, for what medical problem have you diagnosed for? | 1. HTN 2. DM 3. Cancer 4. If any other, specify ______ |  |

**Section IV: Dietary Assessment (24-hour dietary recall method)**

| 24-hour recall | | | |
| --- | --- | --- | --- |
| Interview date: | | | Day food eaten: |
| Time | Place eaten | Food or drink, description, and cooking method | |
|  |  |  | |
|  |  |  | |
|  |  |  | |
|  |  |  | |
|  |  |  | |
|  |  |  | |
|  |  |  | |
|  |  |  | |
|  |  |  | |
|  |  |  | |
|  |  |  | |
|  |  |  | |
|  |  |  | |
|  |  |  | |
|  |  |  | |
|  |  |  | |
|  |  |  | |
|  |  |  | |
|  |  |  | |

**Minimum Dietary Diversity score for Women, MDDS-W model questionnaire**

|  | **Food categories** | **Description (Consumed)** | Yes = 1 No= 0 |
| --- | --- | --- | --- |
| 401 | Foods made from grains | Porridge, bread, rice, pasta/noodles or other foods made from grains | ___ yes (1)  ___ no (0) |
| 402 | White roots and tubers and plantains | White potatoes, white yams, manioc/cassava/yucca, cocoyam, taro or any other foods made from white-fleshed roots or tubers, or plantains | ___ yes (1)  ___ no (0) |
| 403 | Pulses (beans, peas and lentils) | Mature beans or peas (fresh or dried seed), lentils or bean/pea products, including hummus, tofu and tempeh | ___ yes (1)  ___ no (0) |
| 404 | Nuts and seeds | Any tree nut, groundnut/peanut or certain seeds, or nut/seed “butters” or pastes | ___ yes (1)  ___ no (0) |
| 405 | Milk and milk products | Milk, cheese, yoghurt or other milk products but NOT including butter, ice cream, cream or sour cream | ___ yes (1)  ___ no (0) |
| 406 | Organ meat | Liver, kidney, heart or other organ meats or blood-based foods, including from wild game | ___ yes (1)  ___ no (0) |
| 407 | Meat and poultry | Beef, pork, lamb, goat, rabbit, wild game meat, chicken, duck or other bird | ___ yes (1)  ___ no (0) |
| 408 | Fish and seafood | Fresh or dried fish, shellfish or seafood | ___ yes (1)  ___ no (0) |
| 409 | Eggs | Eggs from poultry or any other bird | ___ yes (1)  ___ no (0) |
| 410 | Dark green leafy vegetables | List examples of any medium-to-dark green leafy vegetables, including wild/foraged leaves | ___ yes (1)  ___ no (0) |
| 411 | Vitamin A-rich vegetables, roots and tubers | Pumpkin, carrots, squash or sweet potatoes that are yellow or orange inside | ___ yes (1)  ___ no (0) |
| 412 | Vitamin A-rich fruits | Ripe mango, ripe papaya | ___ yes (1)  ___ no (0) |
| 413 | Other vegetables | List examples of any other vegetables | ___ yes (1)  ___ no (0) |
| 414 | Other fruits | List examples of any other fruits | ___ yes (1)  ___ no (0) |

**Required**

| 421 | Any condiments and seasonings, such as: | Ingredients used in small quanttes for ﬂavour, such as chilies, spices, herbs, fish powder, tomato paste, ﬂavour cubes or seeds | ___ yes (1) ___ no (0) |
| --- | --- | --- | --- |
| 422 | Any other beverages and foods (optionally, specify if not listed) | Tea or coﬀee if not sweetened, clear broth, alcohol Pickles, olives and similar | ___ yes (1) ___ no (0) |

**Section V: Anthropometric Measurement**

| **ID** | **Questions** | **Possible Answers** | **Skip** |
| --- | --- | --- | --- |
| 501 | Weight | 1. Measurement 1 ________ 2. Measurement 2 ________ |  |
| 502 | Height | 1. Measurement 1 _________ 2. Measurement 2 _________ |  |

B. Qualitative Methods **Interview Guide**

**B1: In-depth Interview**

Research Title: Nutritional Status and its Determinant Factors among CFSWs in Hawassa City, South Ethiopia

Date (day/month/year): ______________ Name of the interviewer: ________________

Time interview began: _______________ Time interview ended: __________________

Name of the clinic: __________________ Code: ____________

1. What is your age?
2. Tell me about your self, your background, your family, your past, exprience in sex work?
3. Do you have children? If yes, are they living with you? who takes care of them and how much times do you give to your children?
4. What is your reasons or cause to be engage in sex work, how you start sex work?
5. What are the main expenditure of you income? How much do you expend for food?
6. Where do you ussally eat food? Do you cook? With home do you usually eat? How many meals do you usually have a day (frequency)?
7. Have you recently faced any difficulty to get food? Lack of money? Become sick? Loss of appetite due to addictions?
8. What do you think about your body weight? Do you feel or notice any change in your body weight recently?
9. What service do you get from the clinic? Do you have any known medical health problem? If yes what? Have you follow for any treatment?
10. Have you ever obtain any nutrition counselling, nutrition related intervention from service providers from the clinic? If yes, what nutrition counselling have you get?
11. Do you have any other challenges as a sex worker? You can mention it?
12. What do you recommend to improve nutritional status of CFSWs?

**B2. Key Informant Interview**

Research Title: Nutritional Status and its Determinant Factors among CFSWs in Hawassa City, South Ethiopia

Date (day/month/year): ______________ Name of the interviewer: ________________

Time interview began: _______________ Time interview ended: __________________

Name of the clinic: __________________ Position of the interviewer: _______________

Code: ________________

1. What is your responsibility in the clinic?
2. How long have you worked as service provider or peer educator for sex workers in the clinc?
3. Do you receive any training to provide services for commercial female sex wokers? If yes mention training received?
4. How do you explain sex workers who come to your clinic? their personal behaviour, risky sexual behavior, their clients?
5. What service do you provide for sex workers in the clinic?
6. Do they pay for the service they obtain from the clinic? If not, who cover the cost of sex workers in your clinic?
7. What are the common health or medical problem of CFSWs who visits to your clinics?
8. What are the common challenges other than medical problem of CFSWs who visits to your clinics?
9. What counselling program do you have for sex workers? Do you include nutrition counselling in your counselling rogram? Do you have any nutrition intervention for sex workers? If yes, what nutrition counselling/intervention do you provide?
10. What do you recommend to improve nutritional status of CFSWs in your clinic?
